# Graining and Texturing of Metal Surfaces by Picosecond Laser Treatment

**DOI:** 10.3390/ma18071398

**Published:** 2025-03-21

**Authors:** Carmelo Corsaro, Fortunato Neri, Paolo Maria Ossi, Domenico Bonanno, Priscilla Pelleriti, Enza Fazio

**Affiliations:** 1 Department of Mathematical and Computational Sciences, Physics Science and Earth Science, University of Messina, Viale F. Stagno D’Alcontres 31, I-98166 Messina, Italy; carmelo.corsaro@unime.it (C.C.); fneri@unime.it (F.N.); dbonanno@unime.it (D.B.); priscilla.pelleriti@studenti.unime.it (P.P.); 2 Department of Chemical, Biological, Pharmaceutical and Environmental Sciences, University of Messina, Viale F. Stagno D’Alcontres 31, I-98166 Messina, Italy; paolo.ossi@polimi.it

**Keywords:** laser scribing, surface texturing, wettability, roughness

## Abstract

Different approaches have been proposed to control the tribological behavior of materials under different conformal and non-conformal contact conditions with influenced surface texturing. The ever-increasing demand to improve material friction, erosion wear, and adhesion bond strength of coatings is a major concern for the contact interface of surfaces. Laser texturing is considered a promising approach to tuning materials’ tribological properties. The latter are strongly influenced by the texture density and shape imprinted on the engineered materials and vary in dry or lubricating conditions. In this work, the physicochemical properties of picosecond laser-textured surfaces of metallic materials have been systematically analyzed. Specifically, the wettability character of laser-textured materials was correlated with their morphological/compositional features.

## 1. Introduction

Surface topography plays an important role in influencing the tribological properties of materials. Several surface texturing approaches like ultra-short laser texturing have been adopted for surface modification. Laser surface texturing (LST) emerged as an appropriate technique to control the friction and wear behavior of rubbing surfaces during conformal and non-conformal contacts, mechanical interlocking at the coating substrate interface, and creating superhydrophobic surfaces. Compared to conventional approaches, the ablation process with ultra-short laser pulses is substantially melt-free; thus, after the laser treatment, no polishing of the surfaces is required since no pile-up at the edges of the micro-machined material occurs [1].

Several application areas require a high degree of anti-corrosion, anti-friction, and self-cleaning properties of surfaces. The environmentally friendly, fast and highly controllable LST technique allows obtaining superamphiphobic (water and oil repellent) surfaces. The LST treated materials, characterized by a low surface energy and dual micro and nano level roughness, confer very limited mechanical wear sturdiness and long-term durability [2]. LST, being able to create different micro- and nano-textured shapes, has been already used for treating the surfaces of bearings and piston cylinders to overcome friction [3]. In particular, textured cavities allowed the capture of abrasive chips favoring lubrication [4,5].

Despite the potential of the LST technique, several challenges must be addressed to successfully introduce LST on the industrial scale, i.e., process scalability, cost viability, and gas emissions during polymeric-like processing. Together with the laser processing parameters, the LST setup must be optimized (for example, by implementing adaptive optics) to reach a high level of accuracy of the scribing/texturing according to a precise pre-defined design. In addition, the durability and longevity aspects of the hierarchical structure by LST are still a target to reach. For example, when parallel sliding bearings are fabricated, LST was not found to be very useful to generate lift action for the cavitation present in the textured dimples [6]. Furthermore, concerning the micro-lubrication effect on laser-textured steel surfaces, ideal conditions have not yet been achieved since the sliding system forms the boundary lubrication by the oil before the first 50 strokes of sliding. Finally, for thermal sprayed coating systems, the useful adhesion bond strength must be still obtained by LST; in fact, when the cavity size of the texturing pattern increases more than twice the average coating particle size, the bond strength of the resulting coating with the substrate decreases effectively [7].

In this work, a picosecond laser source combined with a galvanometric scanner was used to realize patterned surfaces of different metal foils (brass, molybdenum, nickel, copper and stainless steel) for controlling the material’s wettability/oleophilicity degree. This capability could enable the fabrication of high-performance bearings and other mechanical precision parts [3]. The primary objective was indeed to evaluate the resulting changes in wettability, with particular attention to achieving hydrophilicity or oleophilicity. Several analyses have been conducted including profilometry, scanning electron microscopy and spectroscopic investigations to delve deeper into the sample properties. Literature data do not provide a side-by-side comparison of wettability behavior for different metals under picosecond laser treatments; they only highlight the relevance of such research in advancing surface modification techniques and understanding the responses of a specific material to laser processing.

Furthermore, in this work a correlation between Energy-Dispersive X-Ray Spectroscopy (EDX) and X-ray Photoelectron Spectroscopy (XPS) results is drawn. This is relevant since, in the context of laser-textured surfaces, XPS is more suitable for identifying changes in the surface chemistry (such as oxygen site) that might lead to enhanced hydrophobicity or hydrophilicity. EDX can be useful in identifying any elemental changes in the bulk composition that occur during the texturing process, especially if foreign elements are introduced.

## 2. Materials and Methods

All investigated foils have thicknesses between 0.25 and 0.3 mm. Table 1 reports the elemental atomic fractions estimated by Energy-Dispersive X-Ray Spectroscopy (EDX) on the starting materials.

Distilled water, diiodomethane (Merck KGaA, Darmstadt, Germany, 99% purity) and mineral oil (Total Classic 5, 15W-40—used in a mechanical laboratory) were used as solvents for contact angle measurements.

Laser treatment was performed on all the materials using a micromachining setup with an 8 ps Nd:YVO_4_ laser source (Rapid 10, Coherent). The laser can operate at 532 (frequency doubled) and 1064 nm (fundamental) wavelengths. A galvanometer was used to scan the laser spot across the material foils, changing the scan speed from 50 to 2500 mm/s. The laser beam was focused by the 163 mm focal length of a telecentric objective (Scancube III 10, SCANLAB GmbH, Puchheim, Germany). Laser ablation produced a pattern of overlapping parallel lines in the laser beam direction (each line about 20 μm wide to cover a 6 × 6 mm^2^ area by running a single mark loop). In this study the 532 nm wavelength with a repetition rate of 160 kHz and a power of 1 W has been used.

The surface morphology of the laser-treated specimens was evaluated using (i) a Tencor Alpha-Step^®^ 500 stylus profilometer (KLA—Milpitas, CA, USA), equipped with a 5 μm tip radius with a 60° included angle (0.10 μm lateral resolution; 2.5 nm vertical resolution, scan speed of 10 μm/s, sampling rate of 100 Hz); (ii) a Zeiss (Oberkochen, Germany) Merlin^®^ field emission scanning electron microscope (FE-SEM) equipped with a GEMINI II electromagnetic/electrostatic objective lens system, operating at an accelerating voltage of 20 kV, probe current of 220 pA and detecting secondary electrons (SE). The FE-SEM was equipped with a Bruker EDX (Bruker Nano Analytics, Berlin, Germany) probe allowing the compositional analysis of the studied materials in the considered conditions. The surface composition was analyzed by collecting X-ray photoelectron spectroscopy (XPS) spectra with a Thermo Scientific (Waltham, MA, USA) K-alpha spectrometer (a monochromatic Al-Kα (1486.6 eV) source and a hemispherical analyzer operating in constant analyzer energy mode). Before the acquisition, the LST residual/air contaminations were removed by means of an ion source sputtering (energy 2000 eV and etching time 60 s).

Profilometric measurements have been performed to investigate the change in the root mean square roughness (Rq) of the considered metals as a function of the scan speed. Rq is more sensitive to peaks and valleys than the average roughness (Ra) because it squares the amplitudes before averaging, making it more informative for surfaces with significant variations. Rq, being indeed defined as the root mean square average of the profile heights over the evaluation length (*ℓ*), can be evaluated by acquiring the surface profile Z(x) as follows (ISO 4287 is a standard for geometrical product specifications (GPS) related to surface texture, specifically focusing on the profile method):

(1) $$Rq=\sqrt{\frac{1}{\ell }\int_{0}^{\ell }Z^{2}\left(x\right)dx}$$

The surface wettability was determined by analyzing images of the water drops acquired by a homemade static contact angle (CA) setup, equipped with a microliter syringe driven by a micrometer screw, a white LED lamp (580 lumens at 6500 K), an optical lens (focal length of 16 mm, maximum aperture of 1:1.4 to magnify the picture), and an IDS (IDS Imaging Development Systems GmbH, Obersulm, Germany) U3-3884LE-C-HQ camera (1/1.8” sensor size). For each experiment, a drop (about 2 μL) of the considered solvent was gently released onto the textured material placed on the CA stage. A custom MATLAB^®^ R2022b (Mathworks, Natick, MA, USA) script was written to evaluate the contact angle (CA). The MATLAB procedure plans to track the edge of the drop with active contours (energy minimization) and then to reproduce the drop profile with a circle, or an ellipse. Then, the CA values were indirectly measured by drawing a tangent to the profile at a three-phase chosen contact point. Measurements were performed in triplicates, two months after the texturing to reach the equilibrium conditions [8]. The results will be presented as mean ± standard deviation.

The CA formed by the three-phase separation when a solvent drop spreads over a solid surface depends on the interaction strength and on the surface energy. The lower the contact angle, the higher the adhesion work (W) defined as the work needed to separate two different phases. In detail, for solid (S) and liquid (L) phases, Dupré’s equation can be written as follows [9]:

(2) $$W_{SL}=\sigma_{S}+\sigma_{L}-\sigma_{SL},$$

where $\sigma_{S}$ refers to the solid surface energy (SE), $\sigma_{L}$ to the liquid surface tension and $\sigma_{SL}$ to the solid–liquid interfacial tension. The latter can be estimated by measuring the CA ($\theta_{CA}$) through the Young’s equation:

(3) $$\sigma_{SL}=\sigma_{S}-\sigma_{L}\cos\theta_{CA}$$

Equations (2) and (3) can be combined in the so-called Young–Dupré equation which is at the basis of the majority of physical models [9,10] used to define SE (and its polar and dispersive components, see next) of a solid surface when a solvent drop lies on it:

(4) $$W_{SL}=\sigma_{L}\left(1+\cos\theta_{CA}\right).$$

Concerning surface wettability, the water CA is indeed used to determine if a surface is hydrophilic (CA < 90°) or hydrophobic (CA > 90°). With respect to other solvents, such as mineral oil for mechanical applications, CA and SE evaluation are especially relevant for lubrication processes [11,12].

CA values have been measured for the considered material surfaces, i.e., untextured and textured at the selected scan speeds with three different solvents: (1) water to define the wettability and being important in all applications and for which both the dispersive and the polar components are known; (2) diiodomethane, usually considered the reference nonpolar solvent for which is known the surface tension being only dispersive in nature; (3) mineral oil to evaluate the change in oleophilicity possibly induced by laser texturing of the surfaces of the considered materials. CA measurements were performed two months after the texturing to reach the equilibrium conditions and, especially for the mineral oil case after the drop spreading over the surface. In fact, the problem of oil spreading and CA hysteresis phenomena are challenges to overcome for precise CA determination [13,14]. For this reason, sample surfaces were subjected to a cleaning process to eliminate air contamination before CA measurements.

Measuring CA values for solvents with different polar characters allows evaluating SE components by following the Fowkes model [15] which offers a physical theory describing SE. Starting from the Young–Dupré equation, both solid and surface energies at first approximation are expressed as the sum of polar and dispersive (non-polar) components:

(5) $$\sigma_{S}=\sigma_{S}^{P}-\sigma_{S}^{D}\;,\;\;\;\;\;\;\;\;\;\sigma_{L}=\sigma_{L}^{P}-\sigma_{L}^{D}$$

The theory, by assuming a geometric mean for the considered interactions, describes the adhesion work as follows:

(6) $$W_{SL}=2\left(\sqrt{\sigma_{L}^{D}\sigma_{S}^{D}}+\sqrt{\sigma_{L}^{P}\sigma_{S}^{P}}\right)$$

which combined with Equation (4) gives the following:

(7) $$\sqrt{\sigma_{L}^{D}\sigma_{S}^{D}}+\sqrt{\sigma_{L}^{P}\sigma_{S}^{P}}=\frac{\sigma_{L}\left(1+\cos\theta_{CA}\right)}{2}.$$

Considering a fully non-polar (purely dispersive) liquid of known surface tension (such as diiodomethane for which $\sigma_{L}=\sigma_{L}^{D}=50.8\;$ mN/m), allows for the determination of the dispersive component of the solid SE ($\sigma_{S}^{D}$). Then, these values can be used to obtain the polar component of the solid SE ($\sigma_{S}^{P}$) by measuring the contact angle of a liquid for which both polar and nonpolar components are known such as water ($\sigma_{L}^{D}=21.8\;$ mN/m and $\sigma_{L}^{P}=51.0\;$ mN/m) [16].

## 3. Results and Discussion

### 3.1. Contact Angle and Surface Energy

**Figure 1:**
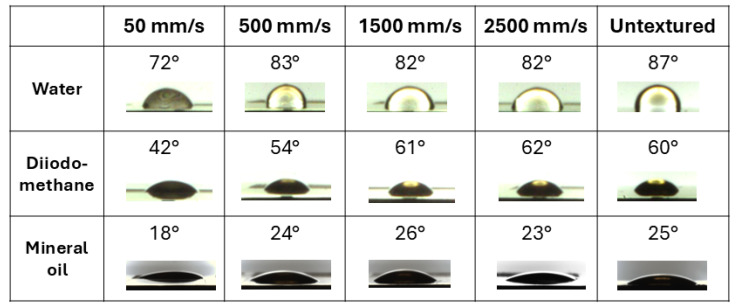
Contact angle images and corresponding values of the solvent drops on brass foils. The standard deviation of CA values is less than 2° in all cases.

**Figure 2:**
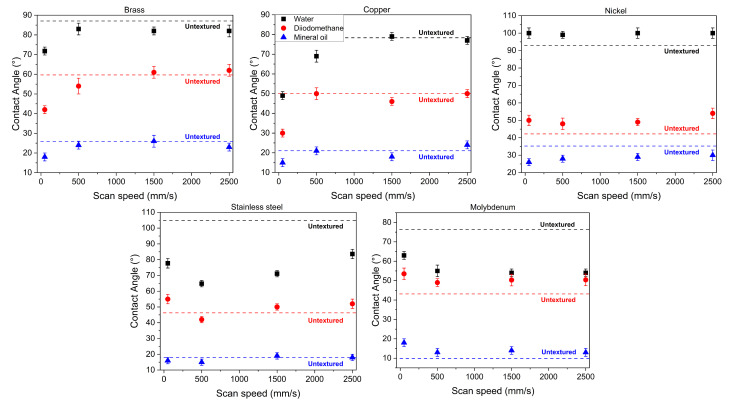
Contact angle values as a function of the laser scan speed using water, diiodomethane and mineral oil as solvents. Dashed lines correspond to CA values for the untextured surfaces.

Figure 1 shows representative acquired drops for the three considered solvents on the surface of untextured and textured brass at scan speeds of 50, 500, 1500 and 2500 mm/s. For all the considered solvents, the drops adhere more on the surface textured at the lowest scan speed whereas upon increasing scan speed, CA values tend towards the value for the untextured case. This is almost a general trend found also for the other investigated metals, as reported in Figure 2.

CA trend for copper resembles that for brass, especially concerning mineral oil as a solvent, whereas CA values for copper are (relatively) smaller than those of brass using water and diiodomethane (Figure 2). In the nickel case, CA values do not change with the surface texturing. Finally, for molybdenum and stainless steel the lowest CA value is observed at 500 mm/s, independent of the solvent and the untextured case presents higher values of water CA. In most cases, CA for the untextured surfaces (dashed lines in Figure 2) represents the asymptotic value at a very high (infinite asymptotically) laser scanning speed. Exceptions are again molybdenum and stainless steel, especially with water as a solvent.

**Figure 3:**
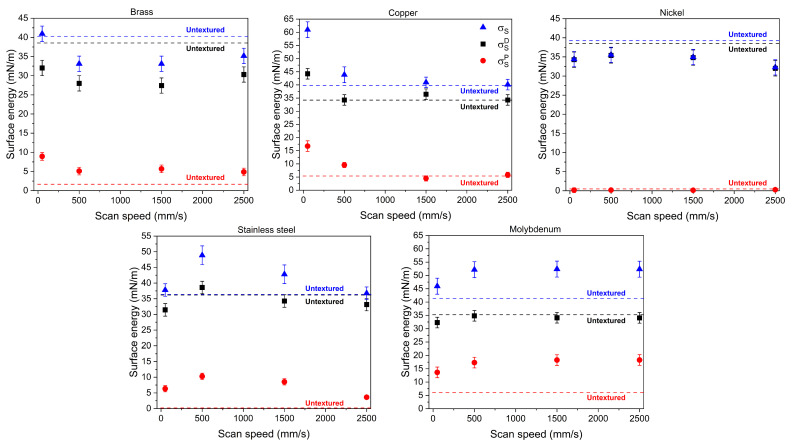
Surface energy (SE) vs. texturing scan speeds for all the studied metal foils. The dispersive (squares) and polar (circles) SE components are shown together with the total surface energy (triangles). Dashed lines correspond to SE values for the untextured surfaces.

Figure 3 shows the calculated SE values (including both polar and dispersive components) as a function of the texturing scan speed.

The nickel SE is fully dispersive and almost unaffected by laser texturing. For brass and copper, SE shows the maximum value (41 and 61 mN/m, respectively) at the lowest scanning speed and a very small (lower than 5 mN/m) polar contribution for the untextured sample. For molybdenum and stainless steel, SE shows a very low (almost zero) polar contribution for the untextured samples and a maximum value (about 50 mN/m) at the scan speed of 500 mm/s.

### 3.2. Roughness

**Figure 4:**
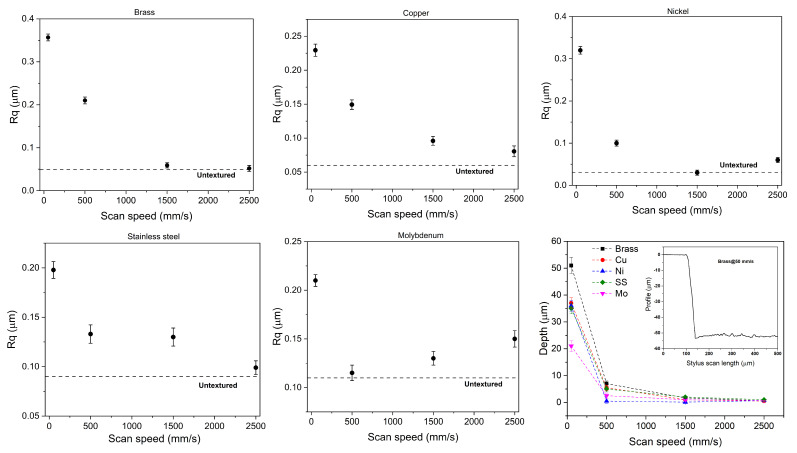
Root mean square roughness vs. texturing scan speeds for all the studied metals. Dashed lines correspond to Rq values for the untextured surfaces. The bottom right panel reports the texturing depth as a function of the scan speed evaluated from acquired profiles (in the inset see the example for brass textured at 50 mm/s).

First, a feeling of the macroscopic effects (on a micrometric scale) of the picosecond laser treatment was gained by carrying out profilometric measurements. Figure 4 shows the measured Rq for all the considered materials as a function of the texturing scan speed. The highest roughness, Rq, occurring at the lowest scan speed (50 mm/s), is ascribed to a more significant accumulation of material and irregularities in the peaks and valleys of the textured surfaces. For brass, copper, nickel and stainless steel, Rq shows an exponential-like trend upon increasing the scan speed. For molybdenum a nearly positive linear trend of Rq vs. the scan speed (from 500 up to 2500 mm/s) was observed as already obtained using a 20 W pulsed nanosecond fiber laser on a Co–Cr–Mo alloy [17]. In addition, profilometric measurements allowed us to estimate the groove depth as a function of the scan speed (bottom right panel of Figure 4). The inset reports the profile acquired for brass textured at 50 mm/s. From the trend in Figure 4, one can observe that at 50 mm/s (i) all metals have the highest depth that tends to zero on increasing the scan speed; (ii) molybdenum grooves have the lowest depth.

**Figure 5:**
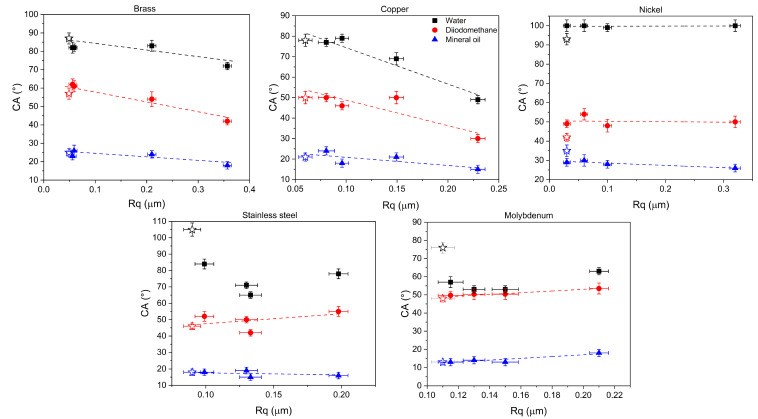
CA vs. Rq for all the considered textured material surfaces. Star symbols correspond to the values for the untextured foil; dashed lines are guides for the eye.

For applications in material science engineering, understanding the correlation between roughness/groove depth and contact angle is relevant. In particular, evaluating CA for different solvents allows us to assess the surface energy and the wettability characteristics. Figure 5 displays CA values as a function of Rq.

In agreement with the Wenzel theory [18], the contact angle increases with roughness for hydrophobic surfaces and decreases with increasing roughness for hydrophilic surfaces. However, the relationship is not straightforward. Indeed, increasing the laser energy density during texturing can lead to a linear increase in the contact angle up to a certain threshold, after which the contact angle stabilizes [19]. Huang et al. [20,21] have also found that increasing surface roughness not always leads to a significant change in contact angle when the laser power is fixed and the scan speed only is varied. This is essentially what can be observed for nickel. For copper and brass, independently from the used solvent, CA decreases upon increasing Rq. For stainless steel, a parabolic relationship has been observed only using water: CA initially decreases, then it increases again. The trend suggests that there is an optimal range of parameters (laser power, scan speed) that minimizes roughness while maximizing wettability [22]. Finally, for molybdenum, CA values remain essentially constant like in the nickel case.

### 3.3. SEM and EDX Analyses

**Figure 6:**
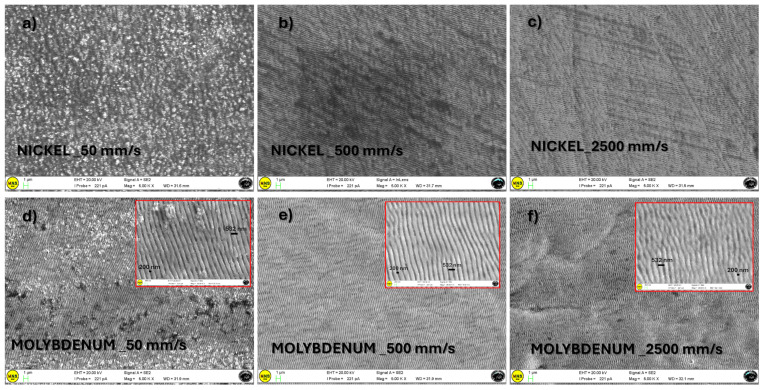
SEM images at different magnifications of nickel (**a**–**c**) and molybdenum (**d**–**f**) samples textured at scan speeds of 50, 500 and 2500 mm/s. The insets of panels (**d**–**f**) show a higher magnification of molybdenum surfaces to highlight LIPSS formation. The reported distance is the laser wavelength.

The 5-micron tip radius used to estimate the samples’ surface roughness is not ideal for capturing the fine details of LIPSS, due to its size and resolution limitations. SEM provides high-resolution images of surface topography, allowing for a detailed visualization of LIPSS. Figure 6 reports SEM images for nickel and molybdenum samples textured at different scanning speeds. In both cases, the surfaces textured at the lowest scanning speed (50 mm/s) display the removal of materials with irregular grain-like structures (on a micrometer scale) of higher roughness [23], confirming the above-discussed Rq trend. The insets for molybdenum at a higher magnification allow us to see the Laser-Induced Periodic Surface Structures (LIPSS), whose periodicity is comparable to the laser wavelength (Low Spatial Frequency LIPSS, LSFL) [24]. The generated ripples are orthogonal to the polarization direction of the laser beam [25].

**Figure 7:**
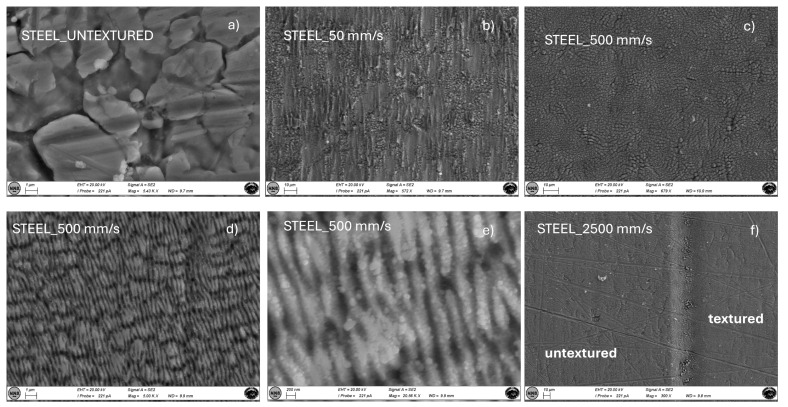
SEM images at different magnifications of stainless steel samples untextured (**a**) and textured at scan speeds of 50 (**b**), 500 (**c**–**e**) and 2500 (**f**) mm/s.

Figure 7 reports SEM images for stainless steel samples textured at different scanning speeds, compared with the untextured case. In particular, Figure 7f allows for a direct comparison of high-speed texturing (2500 mm/s) with the untextured case. In this latter case, no significant morphological changes can be envisaged. The surface morphology of the sample textured at 500 mm/s is reported at different magnifications (Figure 7c–e). Also, in this case, the lower the texturing speed, the higher the surface irregularity and the corresponding average roughness. The highest magnification allows for the observation of LIPSS which shows nanosized grains as interstitial products. These can be the cause of the parabolic behavior observed in Figure 5. The low thermal conductivity of stainless steel (about 16 ${\mathrm{Wm}}^{-1}$$K^{-1}$ at 20 °C) may favor localized overheating with reduced heat transfer efficiency, which results in uneven cavity sizes and shapes due to inconsistent melting and vaporization rates during the laser processing.

**Figure 8:**
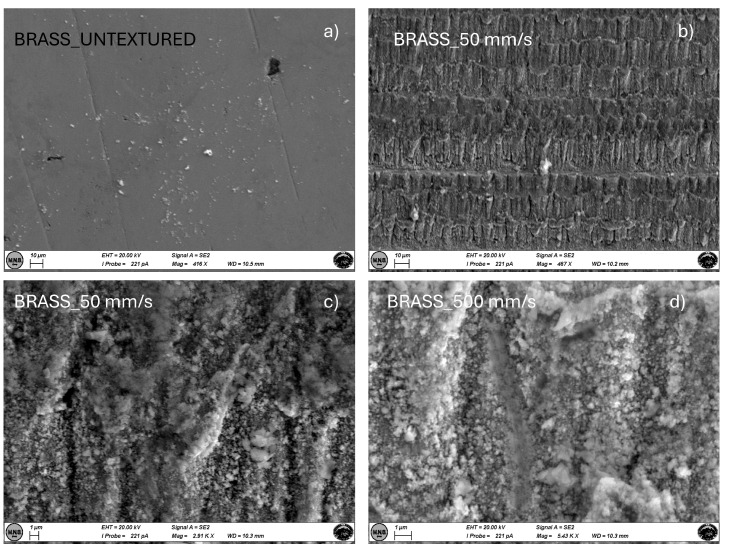
SEM images at different magnification of brass samples untextured and textured (**a**) at scan speeds of 50 (**b**,**c**) and 500 (**d**) mm/s.

**Figure 9:**
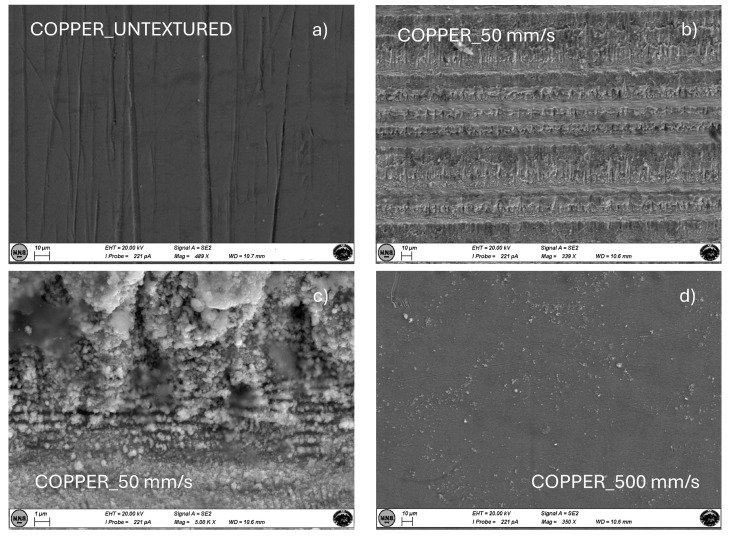
SEM images at different magnifications of copper samples untextured (**a**) and textured at scan speeds of 50 (**b**,**c**) and 500 (**d**) mm/s.

Finally, Figure 8 and Figure 9 show SEM images for brass and copper samples, respectively, textured at scan speeds of 50 and 500 mm/s, compared with the untextured case. For both materials, the effect of laser texturing highly influences the surface morphology although LIPSS cannot be observed even at the highest magnification. This could be ascribed to the lower melting temperature of these metals compared to the others. In fact, for brass $T_{M}^{brass}$ = 930 °C, and for copper $T_{M}^{Cu}$ = 1084 °C whereas for nickel $T_{M}^{Ni}$ = 1453 °C, and for stainless steel $T_{M}^{SS}$ = 1375–1530 °C and for molybdenum $T_{M}^{Mo}$ = 2623 °C.

Laser texturing involves high-energy pulses that induce localized heating; hence, understanding enthalpy helps predict these thermal effects. In detail, monitoring enthalpy (1) ensures efficient use of laser energy, minimizing heat-affected zones and avoiding undesirable effects like material warping or excessive ablation; (2) allows to achieve specific functional properties, such as hydrophobicity, hydrophilicity, or enhanced optical absorption. For instance, laser-induced periodic surface structures (LIPSS) rely on controlled thermal input to create nanoscale patterns that modify wettability or optical characteristics [26]. Moreover, the enthalpy change during the laser treatments is influenced by the transition from solid to vapor or plasma, depending on the laser parameters such as fluence and pulse duration [27]. Therefore, the final texturing effects on metals are influenced by the different enthalpy values of the metals and also by the enthalpic changes occurring during the laser treatment. To this end, it is useful to take into account the enthalpy values of the investigated materials. Brass has a melting enthalpy which varies depending on the alloy composition (e.g., zinc content), exhibiting complex absorption characteristics due to its alloy nature. So, its absorption depends on surface conditions but generally shows high reflectivity in the visible and near-infrared fields.

In a similar way, stainless steel alloys have varying enthalpies depending on their composition and show increasing absorption with rising temperatures due to interband transitions and Fermi band broadening. Moreover, surface roughness also significantly impacts the optical absorption response. Nickel has a melting enthalpy of about 17.48 kJ/mol and a significant interband absorption due to its electronic structure. Its absorptivity increases with temperature as conduction electron density changes with thermal expansion. Molybdenum has a high melting enthalpy of about 36 kJ/mol, reflecting its strong metallic bonding. Molybdenum’s optical properties are influenced by its high melting point (2623 °C) and thermal stability. The absorptivity is low at room temperature but increases at elevated temperatures due to thermal effects. Finally, copper has a melting enthalpy of around 13 kJ/mol and shows high reflectivity in visible light but absorbs more in the infrared. Its absorptivity increases slightly with temperature due to changes in electronic density. Ultimately, the material enthalpy significantly affects the investigated laser-textured surfaces by influencing heat accumulation, meltpool dynamics, microstructure evolution, and mechanical properties as already reported [28,29].

For each material characterized by specific physico-chemical properties, optimizing laser texturing parameters is essential to maximize the material removal rate (MRR), while balancing precision and surface quality [30]. The laser patterning using ultrashort laser pulses involves several interdependent parameters, including laser power, pulse overlap, repetition rate, and scan speed. These parameters collectively influence the interaction between the laser and the material, determining the precision and morphology of the patterned structures.

Laser power determines the energy delivered to the material per pulse:

(8) $$P_{peak}=\frac{E_{pulse}}{\tau }$$

where the energy per pulse $E_{pulse}$ is expressed in joules and the pulse duration τ in seconds. The average power is given by the following:

(9) $$P_{avg}=E_{pulse}\times f$$

where f is the repetition rate in Hz. Higher power increases ablation depth and MRR but can lead to overheating or unwanted thermal effects if not carefully controlled. For ultrashort pulses, the energy is confined to a small region, minimizing thermal diffusion and enabling precise structuring [31].

Pulse overlap is defined by the ratio of the laser spot size and the distance between successive pulses during scanning. So, the overlap degree (OD) between successive laser pulses can be defined as [32,33]:

(10) $$OD=\left(1-\frac{V_{s}}{f\times d}\right)\times 100$$

where $V_{s}$ is the laser scan speed, *f* is the repetition rate and *d* is the laser spot diameter. With f = 160 kHz and d = 23 μm, the pulse overlap ranges from about 99% at 50 mm/s to 32% at 2500 mm/s. High overlap improves uniformity in patterning but may lead to excessive heat accumulation, potentially causing defects or material damage. Conversely, low overlap can result in discontinuities in the patterned structures [34]. In addition, higher repetition rates (frequency of laser pulses) can increase the processing speed but may also lead to heat buildup if the cooling time between pulses is insufficient. This is particularly important in materials with low thermal conductivity (not explored here). Finally, the scan speed determines how quickly the laser moves across the material surface. The increase in the scan speed decreases the overlapping or number of pulses per shot. Therefore, the laser radiation-material interaction time is defined by the pulsed length: faster scan speeds reduce thermal accumulation but may require higher pulse energy or repetition rates to maintain sufficient energy deposition for pattern formation [30,31,35,36]. Specifically, the increase in the melted material with the reduction in the scan speed is due to the accumulation of the pulses, which intensifies the energy density at specific locations. This enhances nonlinear absorption processes, such as multiphoton absorption, further increasing localized melting [35,37].

Regarding the investigated materials, copper has lower ablation thresholds compared to brass, allowing for more efficient material removal at lower laser fluences; brass shows a higher roughness (Figure 4) due to its lower melting point and higher thermal conductivity. According to the literature [38], brass tends to have a lower removal rate compared to copper, which can achieve higher removal rates exceeding 40 mm^3^/min under optimal conditions. Overall, optimizing scanning speed is essential for maximizing MRR while balancing precision and surface quality. Laser irradiation creates a dual-scale roughness structure on the sample surfaces (micro-pattern and LIPSS nano-pattern) mainly at the scan speed of 50 mm/s. For all materials, these morphological features appear to be responsible for carbon deposition on the substrate surface (see EDX data in Table 2). Particularly, for brass and copper in the performed study, when the LIPSS nanopattern covers the micropatterned surface, CA values slightly increase. However, the effects are less evident than those obtained by other researchers using a femtosecond laser source [39,40].

Ultrashort laser pulses interact in a very specific way with metals like brass, molybdenum, copper, stainless steel, and nickel due to their distinct electron-lattice dynamics. Metals with high thermal conductivity (e.g., copper) exhibit steep temperature gradients and extremely high cooling rates (up to $10^{13}$ K/s). This minimizes heat-affected zones (HAZ), enabling precise material processing [41]. The refractory metals—nickel and molybdenum—show minimal grain growth during ablation, maintaining structural integrity under ultrashort pulse processing [42].

As evidenced by the achieved results, increasing pulse overlap during texturing can lead to more complex surface morphologies, improving wettability by altering both topography and local chemical composition. Up to here, the changes in contact angle observed in laser-textured metals are attributed to alterations in surface roughness, or morphology [32]. However, since laser texturing enhances the surface area, the resultant wettability could be significantly affected by the chemical changes introduced during the texturing process.

**Figure 10:**
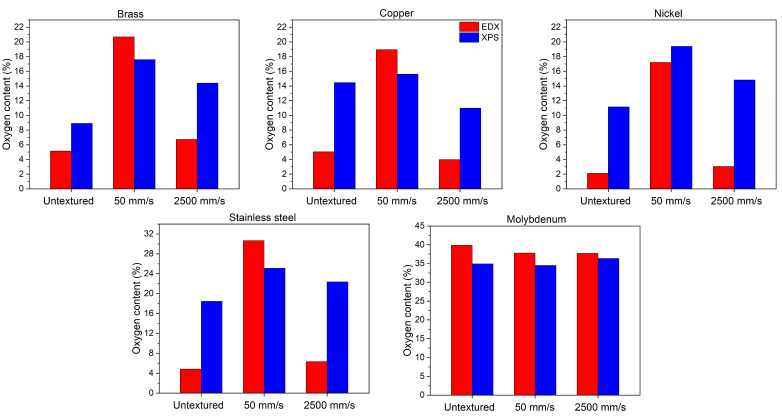
Oxygen percentage content evaluated by EDX and XPS analyses.

EDX measurements have been performed to study the compositional changes induced by laser texturing at different scan speeds. Table 2 reports the percent elemental composition of the studied materials. The lowest scan speed (50 mm/s) allows for more prolonged laser-material interactions, since lower scanning speeds allow consecutive laser pulses to overlap more significantly on the material surface, in turn, increasing the energy deposition per unit area. The increased surface temperatures promote the formation of micro-nanostructures that can trap oxygen. The textured surfaces exhibit enhanced surface area which can further facilitate oxygen absorption from the atmosphere that, together with the temperature increase, facilitates the oxidation process, resulting in a thicker oxide layer on the material surface [3]. When the laser scanning speed exceeds a certain threshold (which changes from 500 mm/s to 2500 mm/s for each metal), the scanning track becomes discontinuous. This leads to insufficient melting of the metal, resulting in defects such as unmelted particles. These defects can inhibit the incorporation of oxygen into the molten pool since there is not enough time for the surrounding material to completely melt and interact with any available oxygen. The rapid cooling does not allow for adequate interaction between the melt pool and oxygen from the atmosphere, which could otherwise lead to oxidation. Consequently, even if oxygen is present in the environment, its effect may be mitigated due to the thermal dynamics. In the samples here investigated, as evidenced by the oxygen amount estimated by EDX (Figure 10), this process is more pronounced for molybdenum than for the other analyzed metals [43,44]. In addition, the purity of the materials or the alloy composition significantly affects the diffusivity values [45,46,47]. Overall, the lack of significant changes in oxygen levels suggests that faster processing limits oxidation reactions within the melt pool.

Finally, as expected, X-ray photoelectron spectroscopy (the technique is particularly sensitive to the near-surface region, with an information depth typically around 10 nm) shows that the oxygen content remains almost unchanged at the different scan speeds for copper, stainless steel and molybdenum with respect to the untextured samples. A measurable rise in the oxygen content, due to surface oxidation processes, likely occurred during and after the laser treatment and was observed for brass and nickel. When metals are exposed to air, they always tend to form an oxide passivation layer [48,49,50,51]. For copper, stainless steel, and molybdenum, with different oxidation behaviors due to their distinct chemical compositions and their ability to form protective oxide layers, the rapid ablation process dominates over slower oxidation kinetics under picosecond laser irradiation. Picosecond lasers operate with extremely short pulse durations (on the order of 10^−12^ s), which minimize the thermal diffusion into the material. This results in highly localized energy deposition, usually reducing the likelihood of significant oxidation or chemical changes during the process. The observation that brass and nickel exhibit a measurable rise in oxygen content due to surface oxidation during picosecond laser treatment can be explained by differences in material properties and oxidation behavior under laser irradiation: brass (an alloy of copper and zinc) and nickel have higher tendencies to oxidize under certain conditions compared to pure copper or stainless steel. Nickel forms stable oxides more readily when exposed to high-energy processes like laser irradiation in atmospheric conditions. Stainless steel is resistant to oxidation due to its chromium content, which forms a protective oxide layer. Molybdenum also has a high melting point and forms less reactive oxides under similar conditions.

These considerations highlight the importance of material selection and understanding of surface chemistry in laser processing applications. Further studies using the XPS technique will be conducted to provide detailed insights into the oxidation mechanisms and their dependence on laser parameters and material properties such as laser-induced roughness or morphology. Actually, based on our data, it emerges that both factors—laser induced roughness and oxygen content—play crucial roles in determining the contact angle. However, surface roughness often has a more direct and immediate effect, while changes in oxygen content contribute to longer-term wettability dynamics.

All the above trends and the main different processes involved during LST must be taken into account to optimize laser processing parameters in metal additive manufacturing to achieve the desired material properties avoiding unwanted oxidation effects.

## 4. Conclusions

The possibility to carry out, following a pre-defined design, an efficient laser-texturing of copper, molybdenum, stainless steel, nickel and brass foils was investigated. In particular, the relationships between the material surface wettability and morphology changes were studied. The hydrophobic/hydrophilic character of the samples has been controlled by appropriately modifying the shapes and sizes of the textured patterns. As evidenced by EDX and XPS data, the laser-induced modification of surface wettability cannot be solely correlated with surface morphology. Ultimately, compositional changes during and after the laser texturing must also be considered to tune the wettability properties of the materials. The textured surfaces allow for better lubrication retention, which minimizes wear and extends the lifespan of components. The micro-patterns created by the picosecond laser texturing could improve the mechanical interlocking between surfaces and adhesives. This improvement is attributed to both the increased surface area and the presence of favorable surface oxides (as observed in this study) as well as to the ability of laser-treated surfaces to retain lubricants better than non-textured surfaces. In addition, textured patterns can help distribute stress more evenly across the surface, reducing localized wear and prolonging the life of coatings, as observed [52,53,54,55,56]. Finally, for the chosen laser processing parameters, the oleophilicity degree of the metal foils was not significantly changed. In the future, to overlap this drawback, the laser-induced roughened surface (that increases the surface area and alters the interaction between the surface and liquids) combined with chemical treatments could effectively be used to enhance the metal oleophilic character, opening up new possibilities for applications requiring specialized surface properties, particularly in industries where oil management is essential.

## Figures and Tables

**Table 1:** Elemental percent composition from EDX analyses of the starting materials.

	Copper	Brass	Molybdenum	Nickel	Stainless Steel
C	26.24%	24.23%	25.61%	19.30%	29.72%
O	5.03%	5.14%	39.89%	2.11%	4.84%
Cu	68.73%	45.44%			
Zn		25.19%			
Mo			27.11%		
N			5.43%		
S			1.95%		
Ni				78.59%	5.08%
Fe					48.14%
Cr					12.22%

**Table 2:** Percent elemental composition (%) from EDX analysis of the studied metal foils.

		Untextured	50 mm/s	500 mm/s	1500 mm/s	2500 mm/s
Brass	Cu	45.44	24.12	42.86	43.22	43.44
	Zn	25.19	15.49	23.77	23.77	24.12
	C	24.23	39.72	25.19	25.27	25.70
	O	5.14	20.68	8.18	7.74	6.74
Copper	Cu	68.73	46.62	70.53	71.97	77.19
	C	26.24	34.43	24.18	24.10	18.84
	O	5.03	18.95	5.29	3.93	3.97
	Mo	27.11	26.94	25.63	30.15	28.04
	N	5.43	0.00	4.96	0.00	0.00
Molybdenum	S	1.95	2.34	1.99	2.47	2.30
	C	25.61	32.93	27.47	32.41	31.95
	O	39.89	37.79	39.95	34.96	37.70
	Ni	78.59	43.51	75.05	79.41	74.64
Nickel	C	19.30	39.28	21.43	17.71	22.32
	O	2.11	17.21	3.52	2.88	3.04
Stainless steel	Fe	48.14	27.71	41.35	43.31	43.51
	Cr	12.22	7.47	10.96	11.23	11.20
	Ni	5.08	2.80	4.24	4.49	4.39
	C	29.72	31.37	31.00	33.50	34.56
	O	4.84	30.66	12.45	7.48	6.34

## Data Availability

The original contributions presented in this study are included in the article. Further inquiries can be directed to the corresponding author.

## Abbreviations

The following abbreviations are used in this manuscript:

LIPSS	Laser Induced Periodic Surface Structure
CA	Contact Angle
SE	Surface Energy
